# MHD flow of time-fractional Casson nanofluid using generalized Fourier and Fick's laws over an inclined channel with applications of gold nanoparticles

**DOI:** 10.1038/s41598-022-21006-9

**Published:** 2022-10-17

**Authors:** Jamal Shah, Farhad Ali, Naveed Khan, Zubair Ahmad, Saqib Murtaza, Ilyas Khan, Omar Mahmoud

**Affiliations:** 1 Department of Mathematics, City University of Science and Information Technology, Peshawar, 25000 Khyber Pakhtunkhwa Pakistan; 2 Department of Mathematics, College of Science Al-Zulfi, Majmaah University, Al-Majmaah, 11952 Saudi Arabia; 3 Petroleum Engineering, Faculty of Engineering and Technology, Future University in Egypt, New Cairo, 11835 Egypt

**Keywords:** Health care, Applied mathematics

## Abstract

Gold nanoparticles are commonly used as a tracer in laboratories. They are biocompatible and can transport heat energy to tumor cells via a variety of clinical techniques. As cancer cells are tiny, properly sized nanoparticles were introduced into the circulation for invasion. As a result, gold nanoparticles are highly effective. Therefore, the current research investigates the magnetohydrodynamic free convection flow of Casson nanofluid in an inclined channel. The blood is considered as a base fluid, and gold nanoparticles are assumed to be uniformly dispersed in it. The above flow regime is formulated in terms of partial differential equations. The system of derived equations with imposed boundary conditions is non-dimensionalized using appropriate dimensionless variables. Fourier's and Fick's laws are used to fractionalize the classical dimensionless model. The Laplace and Fourier sine transformations with a new transformation are used for the closed-form solutions of the considered problem. Finally, the results are expressed in terms of a specific function known as the Mittag-Leffler function. Various figures and tables present the effect of various physical parameters on the achieved results. Graphical results conclude that the fractional Casson fluid model described a more realistic aspect of the fluid velocity profile, temperature, and concentration profile than the classical Casson fluid model. The heat transfer rate and Sherwood number are calculated and presented in tabular form. It is worth noting that increasing the volume percentage of gold nanoparticles from 0 to 0.04 percent resulted in an increase of up to 3.825% in the heat transfer rate.

## Introduction

Over the last decade, nanotechnology has been a hot topic. Material science and biomedicine are the two significant areas of nanoparticle application. Researchers are working on the rapidly growing subject of nanotechnology to understand how to modify matter at the molecular and atomic levels. Research in nanomedicine has become one of the most significant areas of nanotechnology. Since it is indisputably advantageous to modern medicine^1–3^. It is presently concentrating on developing novel technologies to prevent, diagnose, and treat various diseases. Nanomaterials are very effective in killing cancer cells and are now undergoing clinical trials. Nanomaterials are very effective in killing cancer cells and are now undergoing clinical trials. The results are so promising that nanomaterials may become a viable alternative to traditional cancer therapy, especially their capacity to target cancer cells directly and give detailed imaging of tissues, simplifying subsequent therapy planning. Gold metallic nanoparticles are useful in various biomedical applications because of their microscopic size and stability. Bhatti et al^4^ investigated hybrid nanofluid flow with Tantalum $\left( {Ta} \right)$ and Gold $\left( {Au} \right)$ nanoparticles under magnetic effects. They found that magnetic parameter enhances the flow distribution. Saeed Dinarvand et al^5^ examined the stagnation-point boundary layer flow of cuO-Ag/water hybrid nanofluid. In this study, he found that the thermal characteristic of hybrid nanofluid is higher in comparison to the base fluid and fluid containing single nanoparticles. Mousavi et al^6^ investigated the two-dimensional Casson fluid flow of hybrid nanofluids over a stretching sheet. The magnetic field was observed to be normal to the sheet up the velocity profile into the hydrodynamic boundary layer. Dinarvand et al^7^ examined hybrid nanofluid, implying a spinning disk with low to high non-alignments. Their study observed that the second nanoparticle’s mass enhancement results in the amplification of heat transfer.

Gold nanoparticles (GNPs) are a good choice for the treatment of different cancerous cells. Gold nanoparticles are the most significant light-shedding substance in biomedical sciences. The study of gold nanoparticles has recently gotten much attention from researchers because of their structure, form, low toxicity, and excellent compatibility with the human body. Cancer cells were stymied and killed using a unique form of a nanoparticle. Among them, the gold nanoparticle had a particular job. A special type of nanoparticle was utilized to stumble and kill cancer cells. Imtiaz et al^8^ investigated blood flow with a suspension of gold nanoparticles in a vertical tube. During their study, when compared to normal blood, the addition of 0.04-unit gold nanoparticles increased the heat transmission rate by 4%. Aman et al^9^ investigated the effect of gold nanoparticles on mixed convection flow with MHD. Alam et al^10^ studied heat transfer of blood with gold nanoparticles in the presence of magnetic dipole. It was observed that velocity and temperature decrease when ferromagnetic parameter and Prandtl number increase.

The study of the non-Newtonian fluids model has acquired much interest in recent decades because of its applications in industries, engineering, and medicine. Non-Newtonian fluids, such as mud, blood, paint, and polymer solutions are all examples. Due to the complexity of non-Newtonian fluid mechanics, no one model has been able to capture all of its features. A non-Newtonian fluid is the Casson fluid. In the Casson Model, shear thinning, yield stress, and high shear viscosity are all properties of a fluid model^11^. Gowda et al^12^ studied the dynamics of thermal Marangoni stagination point flow in dusty Casson nanofluids. Jyothi et al^13^ explored the squeezing flow of Casson hybrid nanofluids between parallel plates. Shankaralingappa et al^14^ described the influence of sodium Alginate-based Casson nanofluids over a stretching sheet. The modelling and theoretical investigation of Casson nanofluids flow with the influence of magnetic field and chemical reaction explicated by Rivi Kumar^15^. Bhatti et al^16^ investigated natural convection non-Newtonian EMHD dissipative flow through a micro channel. Bhatti et al^17^ studied numerically the flow of hybrid nanofluid through a porous medium. They have chosen water as a base fluid and studied the effect of Cobalt oxide $\left( {Co_{3} O_{4} } \right)$ and Graphene $\left( {Go} \right)$. Qing et al^18^ discussed the thermal assessment of sutterby nanofluid over an axially starched cylinder and obtained the numerical solutions using the shooting method of the involved equations.

Heat transmission is essential in a wide range of biological applications. In the last several decades, there has been a tremendous increase in thermal treatment. Temperature is a critical factor in tissue contact and hyperthermia in living beings. The therapy of hyperthermia involves the application of heat energy to harm cancer^19–22^. Zhao et al^23^ discussed heat and entropy generation in a fluid flow between two rotating disks. Andreozzi et al^24^ proposed hyperthermia therapy via heat transfer. They believed that by employing hyperthermia, tumoral cells would be killed while healthy cells would be saved.

The thought of fractional calculus emerged in 1695. After that, many researchers have given unique definitions of a fractional derivative. Classical derivatives cannot explain some physical and natural phenomena. To depict such a phenomenon, fractional calculus is the best tool to solve these problems. This idea has taken an unusual turn in engineering, biophysics, electrochemistry, mechatronics, and mathematical biology. Different definitions in this field have been suggested by mathematicians, including Rieman-Liouville, Caputo, Atangana-Baleanu, and Caputo-Fabrizio^25–33^. Each definition has its relevance as well as flaws. Many researchers have worked in this field, producing more realistic and generic solutions. Many mathematicians and researchers contributed to the development of derivatives in engineering and mathematical sciences and fractional calculus. The fractional derivative has various practical applications, including geotechnical engineering^34^, quantum physics^35^, and chaotic systems^36,37^. Sheikh et al^38^ studied unsteady MHD flow of Casson fluid in a vertical channel with heat and mass transfer. Their study found that Casson fluid behaves like a Newtonian fluid by increasing the value of the Casson parameter. Ahmad et al^39^ Jeffery nanofluid with joint effects of mass and heat transfer in a horizontal channel. They considered engine oil as a base fluid, and the exact solution was obtained using Laplace and Fourier transform. They concluded that the engine oil efficiency had been improved by 28.24% by adding nanoparticles. Tavazoei et al^40^ discussed the applications of fractional calculus to the propagation of ultrasonic vibrations in human cancellous bone. Ali et al^41^ used the Caputo-Fabrizio time-fractional derivative to analyze the Couette flow of couple stress nanofluids. Magin et al^42^ clarified numerous fractional calculus applications for Bio-Engineers. Moreover, the time-fractional derivative was employed to investigate the tumor dealing model^43^.

Magnetohydrodynamics refers to the study of fluids that conduct electricity in the existence of a magnetic field (MHD). A wide range of bioengineering and medicinal applications are possible with MHD^44^*.* As the first to discover the field of MHD in 1970, Alfven was awarded the Nobel Prize in Physics for his efforts. The magnetic field has broad applications in the field of medical sciences. There are many ways that magnetohydrodynamics (MHD) can be used in biomedical imaging, from the natural magnetization of tissue to fluids that act as contrast enhancers in MTI, CT/X-ray, and optical coherence tomography imaging. Several commercial contrast agents are currently in widespread usage. They also enable better diagnostic imaging (MRI, CT, OCT) and better therapies (targeted drug delivery). Despite the technological hurdles, various medication delivery systems for lung, cancer, and cardiovascular illnesses have been created. Magnetic drug targeting and adjusting blood flow during surgery are examples of these applications^45,46^. Ardahaie et al^47^ investigated the effect of GNPs on blood in the existence of a magnetic field. They calculated the final result using a numerical technique, namely the RK-4 method, and have also shown the impact of several parameters on blood flow. Nazeer et al^48^ investigated the theoretical study of MHD fluid of third-grade fluid in micro channel.

Based on the existing literature, Casson nanofluid flow in an inclined channel with heat and mass transfer has not been studied yet. Therefore, this article aims to make such an attempt. More exactly, in this work, we have considered the Casson nanofluid flow in an inclined channel and the flow is generated due to the oscillation of the plate at $y = d$. The governing equations are transformed to fractional partial differential equations utilizing the Caputo time-fractional derivative definition using extended Fourier and Fick's laws. The Laplace and Fourier sine transforms are used simultaneously to solve the energy and concentration equations, transformed by a newly developed transformation. The resulting general solutions meet all of the requirements imposed on the boundaries, which demonstrates the obtained general solution's validity.

## Mathematical modelling

**Figure 1:**
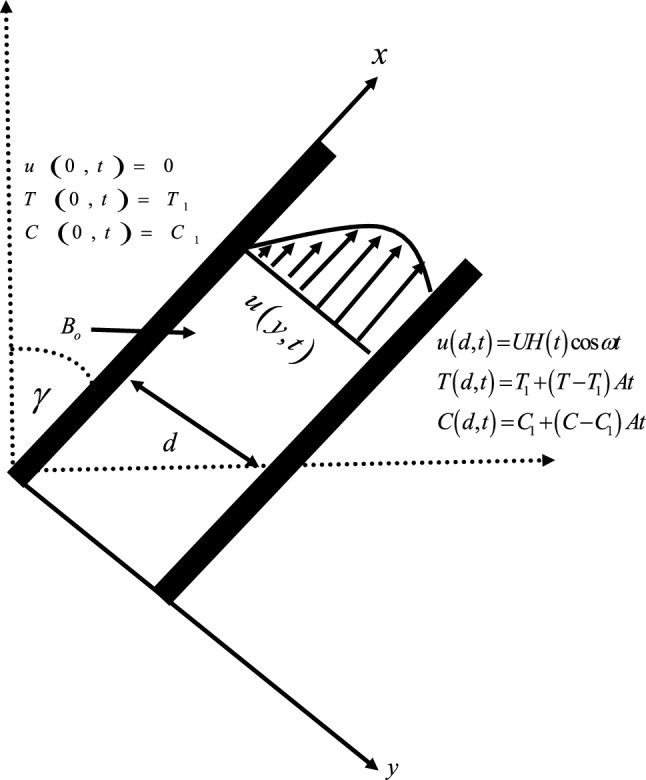
Geometry of the problem.

In the present work, an unsteady blood flow as a Casson nanofluid in an inclined channel is considered. The flow is considered to be in the $x$-direction. The magnetic field is applied transversely to the flow $B_{o}$. Both the fluid and plates are at rest when $t \le 0$ with ambient temperature $T_{1}$ and constant concentration $C_{1}$. However, after a short interval of time $t = 0^{ + }$, the right plate oscillates with the velocity $U$ and frequency $\omega$, and its temperature and concentration are increased to variable temperatures $T_{1} + (T - T_{1} )At$ and variable concentration $C_{1} + (C - C_{1} )At$, respectively as shown in Fig. 1.

In an incompressible Casson fluid, the rheological equation is^50^.

1 $$\tau_{ij} = \left\{ {\begin{array}{*{20}c} {2\left( {\mu_{\gamma } + \frac{{p_{y} }}{{\sqrt {2\pi } }}} \right)e_{ij,} } & {\pi > \pi_{c} ,} \\ {2\left( {\mu_{\gamma } + \frac{{p_{y} }}{{\sqrt {2\pi }_{c} }}} \right)e_{ij,} } & {\pi_{c} < \pi } \\ \end{array} } \right.$$

The velocity field's continuity equation may be approximated using Boussinesq's approximation. $\overrightarrow {V} = (u(y,t),0,0)$ is governed by the partial differential equations given below^51,52^.

2 $$\begin{aligned} \rho_{nf} \frac{\partial u(y,t)}{{\partial t}} = & \mu_{nf} \left( {1 + \frac{1}{\beta }} \right)\frac{{\partial^{2} u(y,t)}}{{\partial y^{2} }} - \sigma_{nf} B_{0}^{2} u(y,t) \\ & + (\rho \beta_{T} )_{nf} g(T - T_{1} )\cos \gamma \, + (\rho \beta_{C} )_{nf} g(C - C_{1} )\cos \gamma , \\ \end{aligned}$$

3 $$(\rho_{Cp} )_{nf} \frac{\partial T}{{\partial t}} = - \frac{\partial m(y,t)}{{\partial y}},$$

4 $$m(y,t) = - k_{nf} \frac{\partial T(y,t)}{{\partial y}}$$

5 $$\frac{\partial C}{{\partial t}} = - \frac{\partial n(y,t)}{{\partial y}},$$

6 $$n(y,t) = - D_{nf} \frac{\partial C(y,t)}{{\partial y}}.$$

Initial and boundary conditions for the problem are specified as:

7 $$\left. \begin{gathered} u(y,0) = 0,\,\,\,\,\,\,\,\,\,T(y,0) = 0,\,\,\,\,\,\,\,\,\,\,\,\,\,C(y,0) = 0,\,\,\,\,\,\,\,\,{\text{for}}\,\,\,t = 0, \hfill \\ u(0,t) = 0,\,\,\,\,\,\,\,\,\,\,T(0,t) = T_{1} ,\,\,\,\,\,\,\,\,\,\,\,C(0,t) = C_{1} ,\,\,\,\,\,\,\,{\text{for}}\,\,\,t > 0, \hfill \\ u(d,t) = UH(t)\cos \omega t,\,\,\,\,\,\, \hfill \\ T(d,t) = T_{1} + (T_{2} - T_{1} )At,\,\,\,\,\,\,\,C(d,t) = C_{1} + (C_{2} - C_{1} )At\,\,\,\,\,\,\,{\text{for}}\,\,\,\,\,t > 0 \hfill \\ \end{gathered} \right\}$$

Following are the nanofluid correlations^53,54^.

8 $$\begin{gathered} \left. \begin{aligned} \rho_{nf} = & \rho_{f} \left( {(1 - \phi ) + \frac{{\rho_{s} }}{{\rho_{f} }}} \right),\,\,\,\,\mu_{nf} = \mu_{f} \left( {\frac{1}{{(1 - \phi )^{2.5} }}} \right),\,(\rho \beta_{T} )_{nf} = (\rho \beta_{T} )_{f} \left( {(1 - \phi ) + \frac{{(\rho \beta_{T} )_{s} }}{{(\rho \beta_{T} )_{f} }}} \right), \\ (\rho \beta_{C} )_{nf} = & (1 - \phi )\left( {(1 - \phi ) + \frac{{(\rho \beta_{C} )_{s} }}{{(\rho \beta_{C} )_{f} }}} \right)\,,(\rho c_{p} )_{nf} = (\rho c_{p} )_{f} \left( {(1 - \phi ) + \frac{{(\rho c_{p} )_{s} }}{{(\rho c_{p} )_{f} }}} \right), \\ \sigma_{nf} = & \sigma_{f} \left[ {1 + \frac{3(\sigma - 1)\phi }{{(\sigma - 2) - (\sigma - 1)\phi }}} \right],\,\,\,k_{nf} = k_{f} \left[ {\frac{{k_{s} + 2k_{f} - 2\phi (k_{f} - k_{s} )}}{{k_{s} + 2k_{f} + \phi (k_{f} - k_{s} )}}} \right], \\ \sigma = & \frac{{\sigma_{s} }}{{\sigma_{f} }},D_{nf} = (1 - \phi )D_{f} \, \\ \end{aligned} \right\}. \hfill \\ \, \hfill \\ \end{gathered}$$

Here $u(y,t)$ is the fluid velocity, $T(y,t)$ and $C(y,t)$ are fluid temperature and concentration, respectively, $\beta$ is a Casson parameter, where $\rho_{nf}$ denotes the nanofluid's density. The dynamic viscosity of a nanofluid is represented by $\mu_{nf}$, while the thermal expansion coefficient and the concentration coefficient are represented by $(\beta_{T} )_{nf}$,$(\beta_{C} )_{nf}$. $B_{0}$ is the magnetic field,$\sigma_{nf}$ signifies the electrical conductivity of nanofluid. $(cp)_{nf}$ denotes the specific heat capacity of nanofluid. The thermal conductivity is shown by $k_{nf}$, while the mass diffusivity is represented by $D_{nf}$. $A$ is constant with dimensions of inverse of $t$.

Introducing the following non-dimensional variables:

9 $$\left. \begin{gathered} v = \frac{u}{U},\,\,\,\,\xi = \frac{y}{d},\,\,\,\,\tau = \frac{v}{{d^{2} }}t,\,\,\,\,\theta = \frac{{T - T_{1} }}{{T_{2} - T_{1} }},\,\,\,\,\Phi = \frac{{C - C_{1} }}{{C_{2} - C_{1} }} \hfill \\ \delta = \frac{md}{{k_{nf} (T_{2} - T_{1} )}},\,\,\,\,\lambda = \frac{nd}{{D_{nf} (C_{2} - C_{1} )}},\,\,\,A = \frac{\upsilon }{{d^{2} }} \hfill \\ \end{gathered} \right\}$$

Equations (2–7) transform to:

10 $$\begin{aligned} \frac{\partial v(\xi ,\tau )}{{\partial \tau }} = & \lambda_{1} (1 + \frac{1}{\beta })\frac{\partial v(\xi ,\tau )}{{\partial \xi^{2} }} - \lambda_{2} Mv(\xi ,\tau ) + \lambda_{3} Gr\theta (\xi ,\tau )\cos \gamma \\ \,\,\,\,\,\,\,\,\,\,\,\,\,\,\,\,\,\,\,\,\,\,\, & + \lambda_{4} Gm\Phi (\xi ,\tau )\cos \gamma , \\ \end{aligned}$$

11 $$\frac{\partial \theta (\xi ,\tau )}{{\partial \tau }} = - \frac{1}{{b_{o} }}\frac{\partial \delta (\xi ,\tau )}{{\partial \xi }},$$

12 $$\delta (\xi ,\tau ) = - \frac{\partial \theta (\xi ,\tau )}{{\partial \xi }}$$

13 $$\frac{\partial \Phi (\xi ,\tau )}{{\partial \tau }} = - \frac{1}{{b_{1} }}\frac{\partial \lambda (\xi ,\tau )}{{\partial \xi }},$$

14 $$\lambda (\xi ,\tau ) = - \frac{\partial \Phi (\xi ,\tau )}{{\partial \xi }}.$$

15 $$\left. \begin{gathered} v(\xi ,0) = 0,\,\,\,\theta (\xi ,0) = 0,\,\,\,\Phi (\xi ,0) = 0\,\,\,\, \hfill \\ v(0,\tau ) = 0,\,\,\,\theta (0,\tau ) = 0,\,\,\,\Phi (0,\tau ) = 0\,\,\,\,\, \hfill \\ v(1,\tau ) = H(t)\cos \omega \tau \hfill \\ \theta (1,\tau ) = \tau ,\,\,\,\Phi (d,\tau ) = \tau \hfill \\ \end{gathered} \right\}.$$

where,

16 $$\left. \begin{aligned} a_{1} = &\, (1 - \phi ) + \frac{{\rho_{s} }}{{\rho_{f} }},\,\,\,a_{2} = (1 - \phi )^{2.5} ,\,\,\,\,a_{3} = 1 + \frac{3(\sigma - 1)\phi }{{(\sigma + 2) - (\sigma - 1)\phi }},\,\,\,\,a_{4} = (1 - \phi ) + \frac{{(\rho \beta_{T} )_{s} }}{{(\rho \beta_{T} )_{f} }},\, \\ \,\,a_{5} = &\, (1 - \phi ) + \frac{{(\rho \beta_{C} )_{s} }}{{(\rho \beta_{C} )_{f} }},\,\,\,a_{6} = (1 - \phi ) + \frac{{(\rho c_{p} )_{s} }}{{(\rho c_{p} )_{f} }},\,\,\,\,a_{\,\,\,7} = \frac{{(k_{s} + 2k_{f} ) - 2\phi (k_{f} - k_{s} )}}{{(k_{s} + 2k_{f} ) + \phi (k_{f} - k_{s} )}},\,\,a_{8} = \left( {1 - \phi } \right) \\ \,\lambda_{\,\,\,1} = &\, \frac{1}{{a_{1} a_{2} }},\,\,\,\lambda_{\,\,\,2} = \frac{{a_{\,\,\,3} }}{{a_{\,\,\,1} }},\,\,\,\lambda_{\,\,\,3} = \frac{{a_{\,\,\,4} }}{{a_{\,\,\,1} }},\,\,\,\lambda_{\,\,\,4} = \frac{{a_{\,\,\,5} }}{{a_{\,\,\,1} }},\,\,\,G\,r = \frac{{\beta_{T} d^{2} g(T_{2} - T_{1} )}}{U\upsilon },\,\,\, \\ G\,m = &\, \frac{{\beta_{C} d^{2} g(C_{2} - C_{1} )}}{U\upsilon },\,\,\,{\text{P}} \,r = \frac{{(\mu cp)_{f} }}{{k_{f} }},\,\,\,S\,c = \frac{\upsilon }{{D_{f} }},\,\,\,b_{o} = \frac{{a_{6} \Pr }}{{a_{7} }},\,\,\,b_{1} = \frac{Sc}{{a_{8} }}. \\ \end{aligned} \right\}$$

The following fractional model is constructed using generalized Fick's and Fourier's laws:

17 $$\delta (\xi ,\tau ) = - {}^{C}\wp_{\tau }^{1 - \alpha } (\frac{\partial \theta (\xi ,\tau )}{{\partial \xi }});\,\,\,\,\,\,\,\,\,\,\,0 < \alpha \le 1,$$

18 $$\lambda (\xi ,\tau ) = -^{C} \wp_{\tau }^{1 - \alpha } (\frac{\partial \Phi (\xi ,\tau )}{{\partial \xi }});\,\,\,\,\,\,\,\,\,\,\,\,\,0 < \alpha \le 1$$

In the above equations ${}^{C}\wp_{\tau }^{1 - \alpha } \{ .\}$ is the time-fractional Caputo derivative delineated by

19 $$\begin{aligned} {}^{C}\wp_{t}^{\alpha } r(y,t) = & \frac{1}{\Gamma (1 - \alpha )}\int_{0}^{t} {\mathop r\limits^{.} (y,t)(t - s)^{ - \alpha } ds} , \\ = & K_{\alpha } (t) * \mathop r\limits^{.} (y,t);\,\,0 \le \alpha \le 1 \\ \end{aligned}$$

The singular power-law kernel is $K_{\alpha } (t) = \frac{{t^{ - \alpha } }}{\Gamma (1 - \alpha )}$.

Likewise,

20 $$\begin{aligned} L\{ K_{\alpha } (t)\} = & \frac{1}{{s^{1 - \alpha } }},\,\,\,\{ K_{{_{1 - \alpha } }} * K_{\alpha } \} (t) = 1,\,\,\,K_{0} (t) = L^{ - 1} \left\{ {\left. \frac{1}{s} \right\} = 1} \right., \\ K_{1} (t) = & L^{ - 1} \{ 1\} = \zeta (t), \\ \end{aligned}$$

The Laplace transform is denoted by $L\{ .\}$. The transform parameter is denoted by $s$, whereas the Dirac's delta distribution is represented by $\delta (.)$. It is simple to demonstrate this.

21 $$\begin{gathered} {}^{C}\wp_{t}^{0} r(y,t) = r(y,t) - r(y,0),\,\,\,\left( {{}^{C}\wp_{t}^{0} r(y,t) = r(y,t)\,if\,r(y,0) = 0\,} \right) \hfill \\ \,{}^{C}\wp_{t}^{1} r(y,t) = \frac{\partial r(y,t)}{{\partial t}}. \hfill \\ \end{gathered}$$

We arrived at the following by using Eqs. (11), (13), (17), and (18), as well as Caputo time fractional operator is defined in Eq. (25).

22 $$\frac{\partial T(\xi ,\tau )}{{\partial t}} = \frac{1}{{b_{0} }}{}^{C}\wp_{\tau }^{1 - \alpha } \left( {\frac{{\partial^{2} T(\xi ,\tau )}}{{\partial \xi^{2} }}} \right).$$

23 $$\frac{\partial \Phi (\xi ,\tau )}{{\partial t}} = \frac{1}{{b_{1} }}{}^{C}\wp_{\tau }^{1 - \alpha } \left( {\frac{{\partial^{2} \Phi (\xi ,\tau )}}{{\partial \xi^{2} }}} \right).$$

To construct the more appropriate version of Eqs. (22) and (23), we revisit the time-fractional integral operator.

24 $$\Im_{t}^{\alpha } r\left( {y,t} \right) = \left( {K_{1 - \alpha } * r} \right)\left( t \right) = \frac{1}{\Gamma \left( \alpha \right)}\int\limits_{0}^{t} {r(y,s)(t - s)^{\alpha - 1} ds.}$$

Which is the inverse of the derivative operator ${}^{C}\wp_{t}^{\alpha } (.).$ We arrived at this conclusion sequentially by applying the properties from Eq. (20).

25 $$\begin{aligned} \left( {\Im_{t}^{\alpha } o{}^{C}\wp_{\tau }^{\alpha } } \right)r\left( {y,t} \right) = &\, \Im_{t}^{\alpha } \left( {{}^{C}\wp_{\tau }^{\alpha } r\left( {y,t} \right)} \right) = \left[ {K_{1 - \alpha } * \left( {K_{\alpha } * \mathop r\limits^{.} } \right)} \right]\left( t \right) \\ = &\, \left[ {\left( {K_{1 - \alpha } * K_{\alpha } } \right) * \mathop r\limits^{.} } \right]\left( t \right) = [1 * \mathop r\limits^{.} ]\left( t \right) = r(y,t) - r(y,0), \\ \end{aligned}$$

which implies

26 $$\left( {\Im_{t}^{\alpha } o{}^{\,C}\wp_{\tau }^{\alpha } } \right)r\left( {y,t} \right) = r\left( {y,t} \right)\,\,\,\,{\text{if}}\;\;r\left( {y,0} \right) = 0.$$

Moreover, using

27 $$\Im_{t}^{1 - \alpha } \mathop r\limits^{.} \left( {y,t} \right) = \left( {K_{\alpha } * \mathop r\limits^{.} } \right)\left( t \right) = {}^{C}\wp_{t}^{\alpha } r\left( {y,t} \right),$$

We get the fractional differential equations below.

28 $${}^{C}\wp_{\tau }^{\alpha } \theta \left( {\xi ,\tau } \right) = \frac{1}{{b_{0} }}\frac{{\partial^{2} \theta \left( {\xi ,\tau } \right)}}{{\partial \xi^{2} }},$$

29 $${}^{C}\wp_{\tau }^{\alpha } \Phi \left( {\xi ,\tau } \right) = \frac{1}{{b_{1} }}\frac{{\partial^{2} \Phi \left( {\xi ,\tau } \right)}}{{\partial \xi^{2} }},$$

## Solution of the problem

To solve the model for the given flow regime, first the energy equation is going to be solved.

### Solution of the energy equation

Using the transformation described below:

30 $$\chi \left( {\xi ,\tau } \right) = \theta \left( {\xi ,\tau } \right) - \xi f(\tau ).$$

Equation (28) takes the following form:

31 $${}^{C}\wp_{\tau }^{\alpha } \chi \left( {\xi ,\tau } \right) - \xi \wp_{\tau }^{\alpha } f\left( \tau \right) = \frac{1}{{b_{o} }}\frac{{\partial^{2} \chi \left( {\xi ,\tau } \right)}}{{\partial \xi^{2} }},$$

With initial and boundary conditions:

32 $$\chi \left( {\xi ,0} \right) = 0,\,\,\,\,\chi \left( {0,\tau } \right) = 0,\,\,\,\,\chi \left( {1,\tau } \right) = 0.$$

For our transformation, we employ Laplace and Fourier sine transforms.

33 $$\mathop \chi \limits^{ - }_{F} \left( {n,s} \right) = \frac{{\left( { - 1} \right)^{n} }}{n\pi }\frac{1}{s}\left\{ {\frac{{s^{\alpha - 1} }}{{s^{\alpha } + \frac{{\left( {n\pi } \right)^{2} }}{{b_{o} }}}}} \right\},$$

Now by applying inverse Laplace transform (LT) and Fourier sine transform, we get

34 $$\chi \left( {\xi ,\tau } \right) = 2\sum\limits_{n = 1}^{\infty } {\frac{{\left( { - 1} \right)^{n} \sin \left( {n\pi \xi } \right)}}{n\pi }\int_{0}^{\tau } {\left( {1 - \tau } \right)E_{\alpha ,\alpha - 1} \left( {\frac{{\left( {n\pi } \right)^{2} }}{{b_{o} }}t^{\alpha } } \right)dt,} }$$

Incorporating Eq. (34) in (30), we get the final closed form solutions in the following form:

35 $$\theta \left( {\xi ,\tau } \right) = \xi f(\tau ) + 2\sum\limits_{n = 1}^{\infty } {\frac{{\left( { - 1} \right)^{n} \sin \left( {n\pi \xi } \right)}}{n\pi }\int_{0}^{\tau } {\left( {1 - \tau } \right)E_{\alpha ,\alpha - 1} \left( {\frac{{\left( {n\pi } \right)^{2} }}{{b_{o} }}t^{\alpha } } \right)dt,} }$$

### Solution of mass equation

Using the transformation described below

36 $$\Psi \left( {\xi ,\tau } \right) = \Phi \left( {\xi ,\tau } \right) - \xi g(\tau ),$$

Equation (29) takes the following form

37 $${}^{C}\wp_{\tau }^{\alpha } \Psi \left( {\zeta ,\tau } \right) - \xi \wp_{\tau }^{\alpha } g\left( \tau \right) = \frac{1}{{b_{1`} }}\frac{{\partial^{2} \Psi \left( {\zeta ,\tau } \right)}}{{\partial \xi^{2} }}.$$

With initial and boundary conditions

38 $$\Psi \left( {\zeta ,0} \right) = 0,\,\,\,\,\Psi \left( {0,\tau } \right) = 0,\,\,\,\,\Psi \left( {1,\tau } \right) = 0.$$

For our transformation, we employ Laplace and Fourier sine transforms.

39 $$\mathop \Psi \limits^{ - }_{F} \left( {n,s} \right) = \frac{{\left( { - 1} \right)^{n} }}{n\pi }\frac{1}{s}\left\{ {\frac{{s^{\alpha - 1} }}{{s^{\alpha } + \frac{{\left( {n\pi } \right)^{2} }}{{b_{1} }}}}} \right\}.$$

Applying inverse Laplace transform (LT) and Fourier sine transform, we get

40 $$\Psi \left( {\xi ,\tau } \right) = 2\sum\limits_{n = 1}^{\infty } {\frac{{\left( { - 1} \right)^{n} \sin \left( {n\pi \xi } \right)}}{n\pi }\int_{0}^{\tau } {\left( {1 - \tau } \right)E_{\alpha ,\alpha - 1} \left( {\frac{{\left( {n\pi } \right)^{2} }}{{b_{1} }}t^{\alpha } } \right)dt,} }$$

Incorporating Eq. (40) in (36), we get the final closed form solutions in the following form:

41 $$\Phi \left( {\xi ,\tau } \right) = \xi g(\tau ) + 2\sum\limits_{n = 1}^{\infty } {\frac{{\left( { - 1} \right)^{n} \sin \left( {n\pi \xi } \right)}}{n\pi }\int_{0}^{\tau } {\left( {1 - \tau } \right)E_{\alpha ,\alpha - 1} \left( {\frac{{\left( {n\pi } \right)^{2} }}{{b_{1} }}t^{\alpha } } \right)dt,} }$$

### Velocity profile

Using Eqs. (10) and (15), the result may be stated as follows using the Laplace and Fourier transforms:

42 $$\begin{aligned} V_{F} (n,s) = &\, \frac{{( - 1)^{n + 1} }}{n\pi }\frac{s}{{s^{2} + \omega^{2} }} + \frac{{( - 1)^{n} }}{n\pi }\left( {\frac{{p_{3} }}{s} + \frac{{p_{2} }}{{s + p_{1} }}} \right) - \frac{{\omega^{2} }}{{s^{2} + \omega^{2} }}\left( {\frac{{p_{3} }}{s} + \frac{{p_{2} }}{{s + p_{1} }}} \right) \\ \,\,\,\,\,\,\,\,\,\,\,\,\,\,\,\,\,\,\,\,\,\, & + \frac{{\lambda_{3} Gr\theta_{F} (n,s)\cos \gamma }}{{s + p_{1} }} + \frac{{\lambda_{4} Gm\Phi_{F} (n,s)\cos \gamma }}{{s + p_{1} }}, \\ \end{aligned}$$

Here

43 $$p = 1 + \frac{1}{\beta },\,\,\,\,p_{1} = \lambda_{2} M + \lambda_{1} p\left( {n\pi } \right)^{2} ,\,\,\,\,p_{3} = 1 - \frac{{\lambda_{1} p\left( {n\pi } \right)^{2} }}{{p_{1} }},\,\,\,\,p_{3} = \frac{{\lambda_{1} p\left( {n\pi } \right)^{2} }}{{p_{1} }}$$

Applying inverse Laplace transform (LT) along with Fourier sine transforms, the final solution is obtained:

44 $$\begin{aligned} v(\xi ,\tau ) = &\, \xi \cos \omega \tau + 2\sum\nolimits_{n = 1}^{\infty } {\left( {\frac{{\left( { - 1} \right)^{n} }}{n\pi }\left( {p_{3} + p_{2} \exp ( - p_{1\tau } )} \right) - \omega \sin \omega \tau * \left( {p_{3} + p_{2} \exp ( - p_{1\tau } )} \right)} \right)} \\ & + 2\lambda_{3} Gr\sum\limits_{n = 1}^{\infty } {\sin (n\pi \xi )\cos \gamma \left( {\frac{{( - 1)^{n} }}{n\pi }\exp ( - p_{1\tau } ) * \int\limits_{0}^{\tau } {(1 - \tau )E_{\alpha ,\alpha - 1} \left( { - \frac{{( - n\pi )^{2} }}{{b_{0} }}t^{\alpha } } \right)} dt + f\left( t \right)} \right)} \\ & 2\lambda_{3} Gm\sum\limits_{n = 1}^{\infty } {\sin (n\pi \xi )\cos \gamma \left( {\frac{{( - 1)^{n} }}{n\pi }\exp ( - p_{1\tau } ) * \int\limits_{0}^{\tau } {(1 - \tau )E_{\alpha ,\alpha - 1} \left( { - \frac{{( - n\pi )^{2} }}{{b_{1} }}t^{\alpha } } \right)dt + g\left( t \right)} } \right)} . \\ \end{aligned}$$

### Limiting case

For $\phi ,\omega \,{\text{and }}\gamma \to 0$, the obtained general solution (44) is reduced to the solution calculated by Sheikh et al^38^. This shows the validity of the present solutions. For details, please see Eq. (40) in^38^.

### Nusselt number

The Nusselt number is an important physical quantity, especially for engineers and industrialists, and it is defined as follows:

45 $$\left. {N_{n} = - \frac{{k_{nf} }}{{k_{f} }}\frac{\partial \theta (\xi ,\tau )}{{\partial \xi }}} \right|_{\xi = 1} .$$

### Sherwood number

The mathematical form of the Sherwood number is defined as follows:

46 $$\left. {S_{n} = - D_{nf} \frac{\partial \Phi (\xi ,\tau )}{{\partial \xi }}} \right|_{\xi = 1} .$$

## Graphical interpretation and discussion

**Figure 2:**
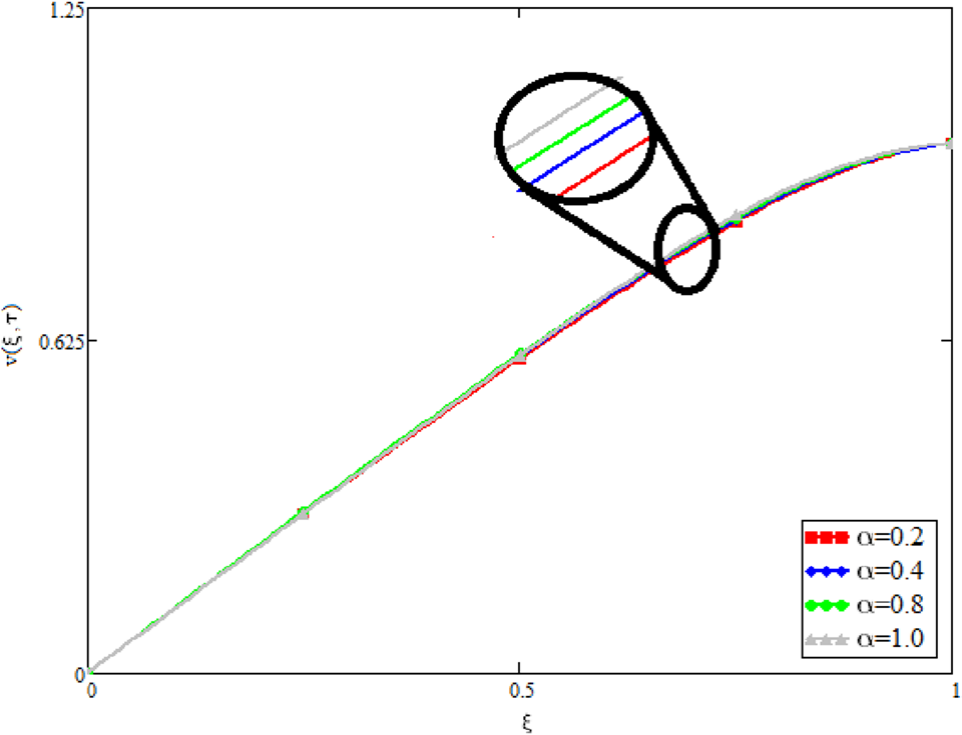
Fractional parameter $$\alpha$$ impact on the velocity profile, when, $$Gr = 0.5$$, $$Gm = 0.5$$,$$\phi = 0.01,$$$$M = 1$$, $$\gamma = \frac{\pi }{4}$$,$$\beta = 1$$, $$\Pr = 22.64$$, and $$Sc = 1.9 \times 10^{4} .$$

**Figure 3:**
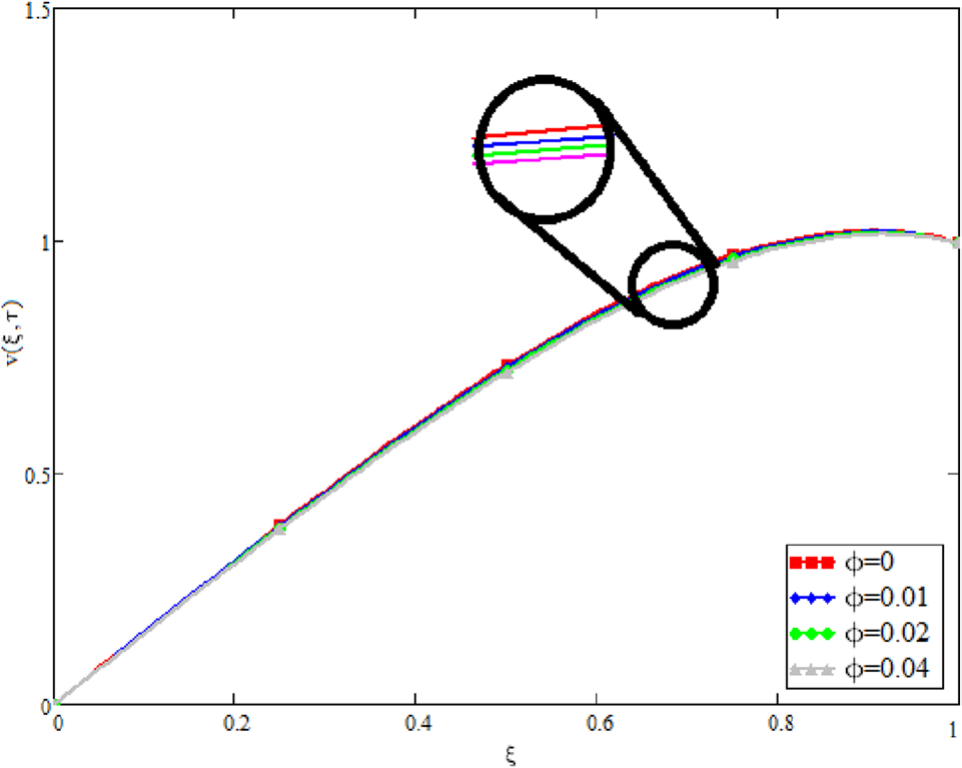
Velocity variation for different values of $\phi$, when, $\alpha = 0.5$, $Gr = 0.5$, $Gm = 0.5$,$M = 1$, $\gamma = \frac{\pi }{4}$, $\beta = 1$, $\Pr = 22.64$, and $Sc = 1.9 \times 10^{4} .$

**Figure 4:**
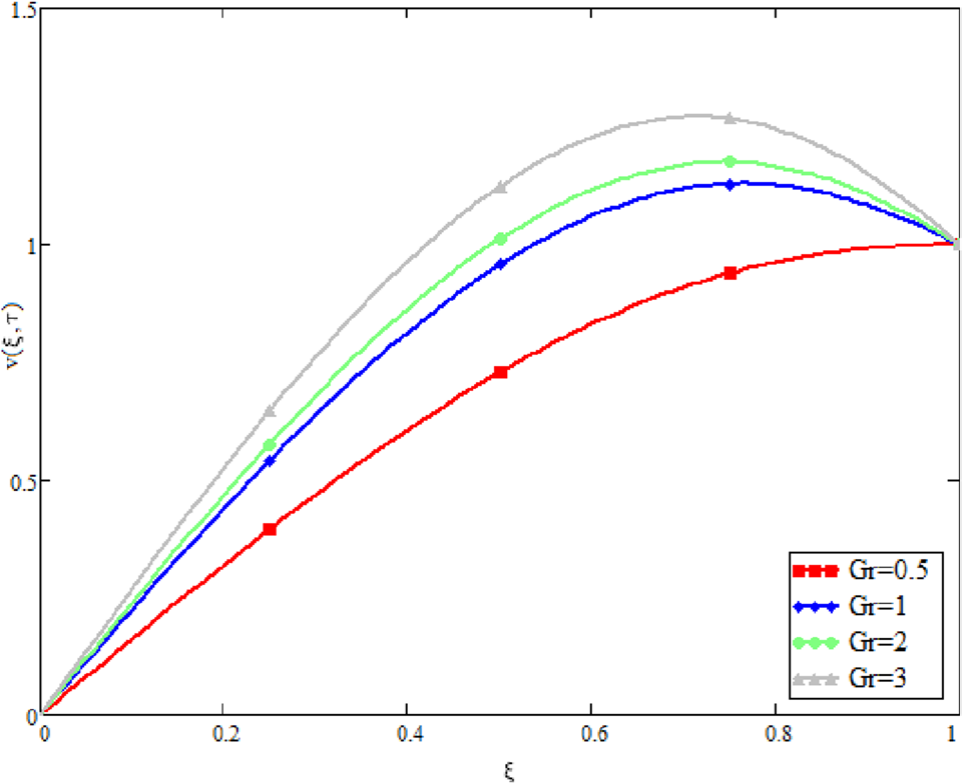
Thermal Grashof number $Gr$ impact on the velocity distribution, when, $\alpha = 0.5$, $\phi = 0.01$, $Gm = 0.5$,$M = 1$, $\gamma = \frac{\pi }{4}$,$\beta = 1$, $\Pr = 22.64$, and $Sc = 1.9 \times 10^{4} .$

**Figure 5:**
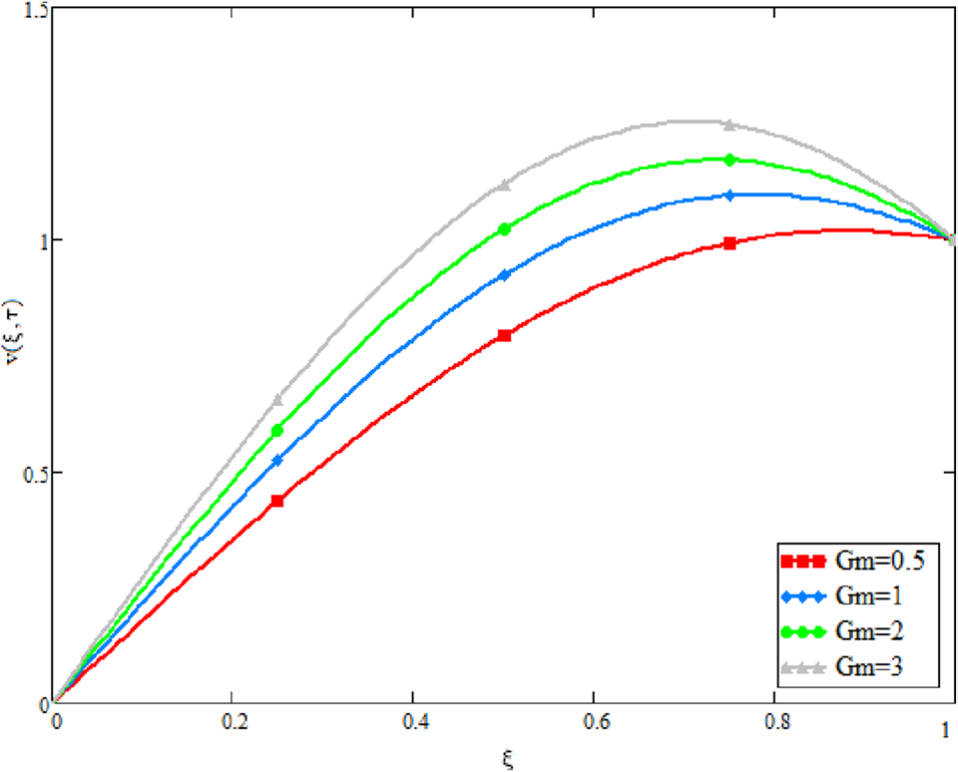
Mass Grashof number $Gm$ impact on the velocity profile, when, when, $\alpha = 0.5$, $Gr = 0.5$, $\phi = 0.01$,$M = 1$ , $\gamma = \frac{\pi }{4}$,$\beta = 1$ , $\Pr = 22.64$, and $Sc = 1.9 \times 10^{4} .$

**Figure 6:**
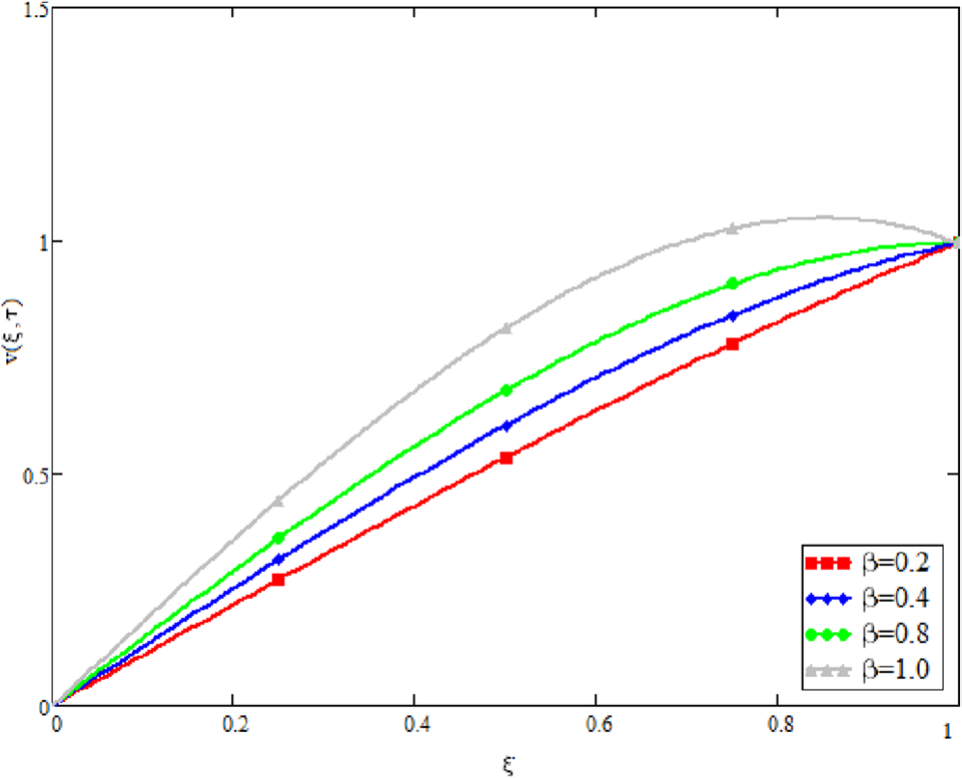
Velocity variation with material parameter $\beta$, when, when, $\alpha = 0.5$, $Gr = 0.5$, $Gm = 0.5$,$\phi = 0.01$, $M = 1$,$\gamma = \frac{\pi }{4}$, $\Pr = 22.64$, and $Sc = 1.9 \times 10^{4} .$

**Figure 7:**
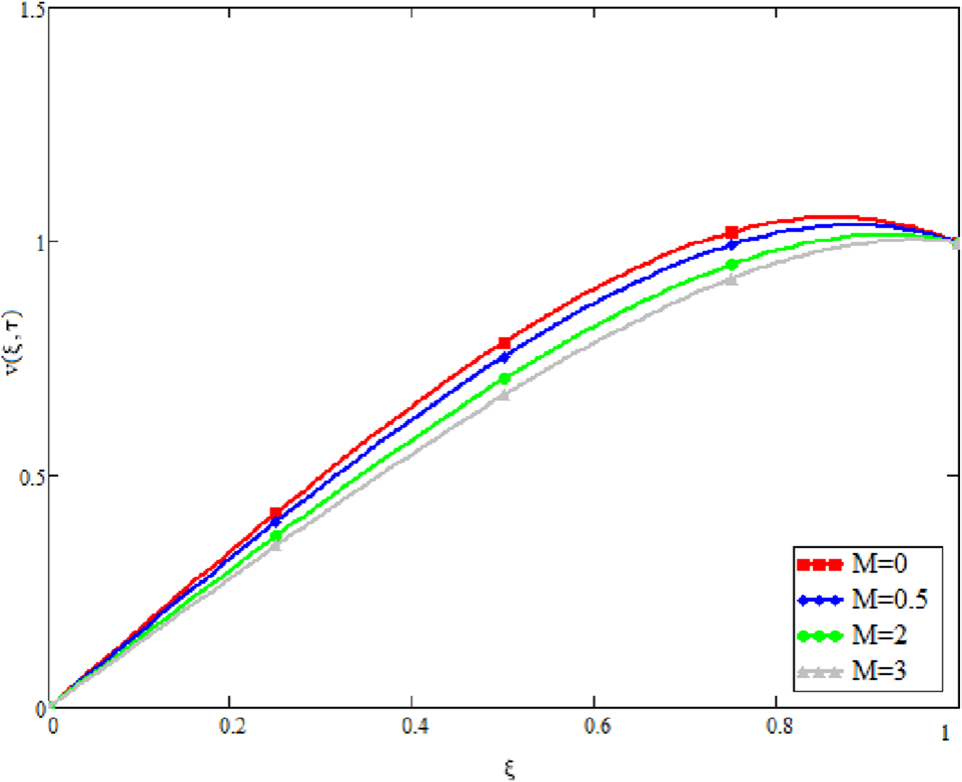
Velocity variation with Hartman number $M$, when, $\alpha = 0.5$, $Gr = 0.5$, $Gm = 0.5$,$\phi = 0.01$, $\gamma = \frac{\pi }{4}$,$\beta = 1$, $\Pr = 22.64$, and $Sc = 1.9 \times 10^{4} .$

**Figure 8:**
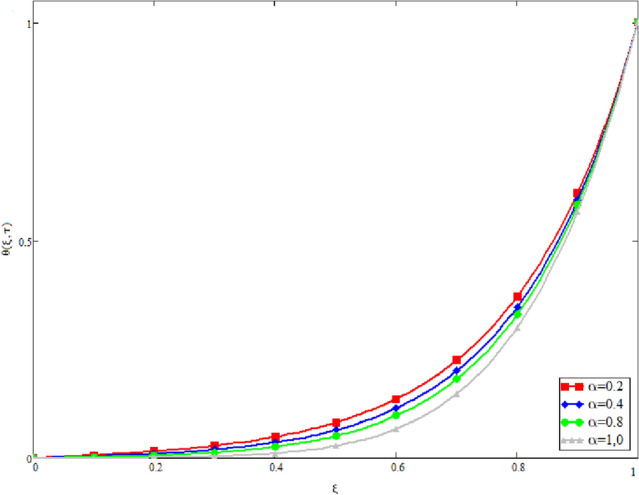
The temperature distribution's derivation of the distinct value of α, when $Gr = 0.5$, $Gm = 0.5$,$M = 1$,$\phi = 0.01$, $\gamma = \frac{\pi }{4}$,$\beta = 1$, $\Pr = 22.64$, and $Sc = 1.9 \times 10^{4} .$

**Figure 9:**
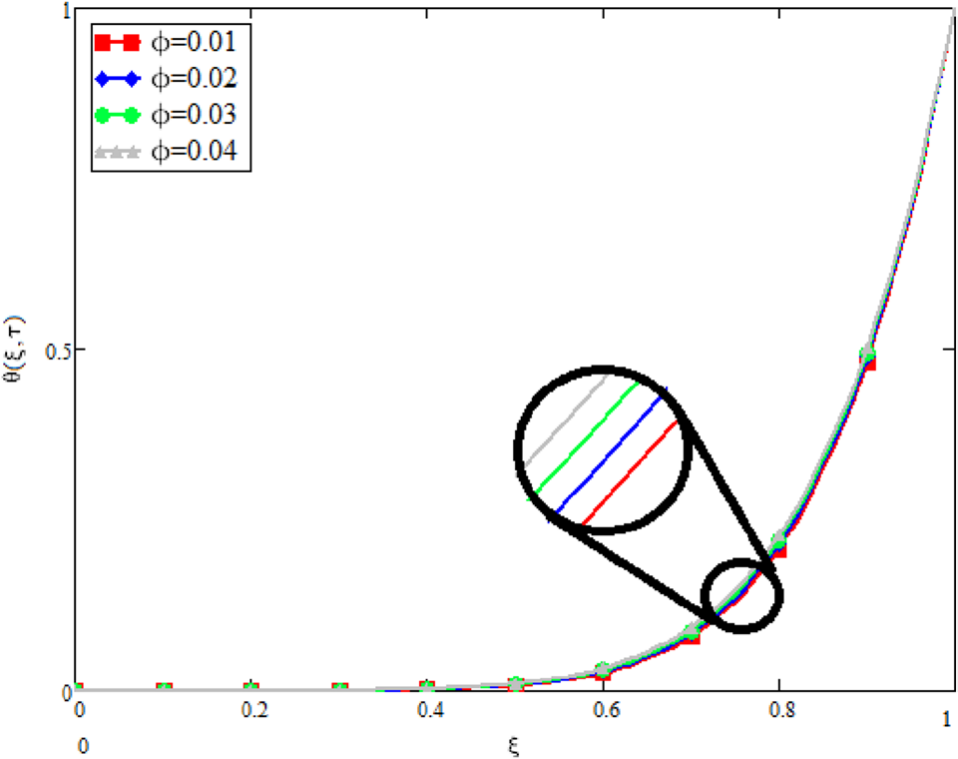
Impact on temperature profile for different values of $\phi$, when $\alpha = 0.5$, $Gr = 0.5$, $Gm = 0.5$,$Gm = 0.5$, $\gamma = \frac{\pi }{4}$,$\beta = 1$, $\Pr = 22.64$, and $Sc = 1.9 \times 10^{4} .$

**Figure 10:**
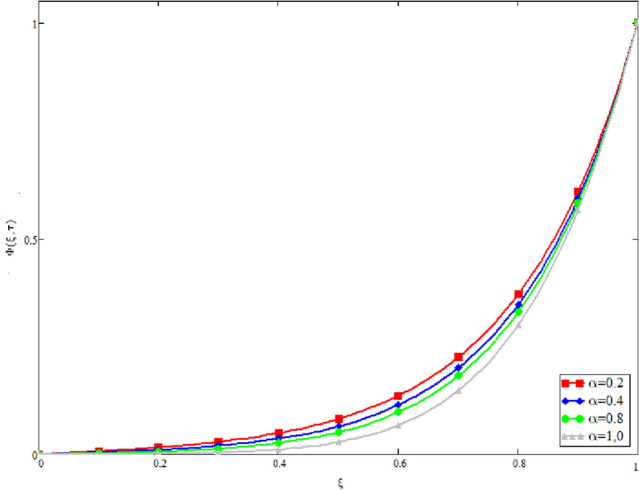
Variation in a profile of concentration for varying values of $\alpha$, when $Gr = 0.5$, $Gm = 0.5$,$M = 1$,$\phi = 0.01$, $\gamma = \frac{\pi }{4}$,$\beta = 1$, $\Pr = 22.64$, and $Sc = 1.9 \times 10^{4} .$

**Figure 11:**
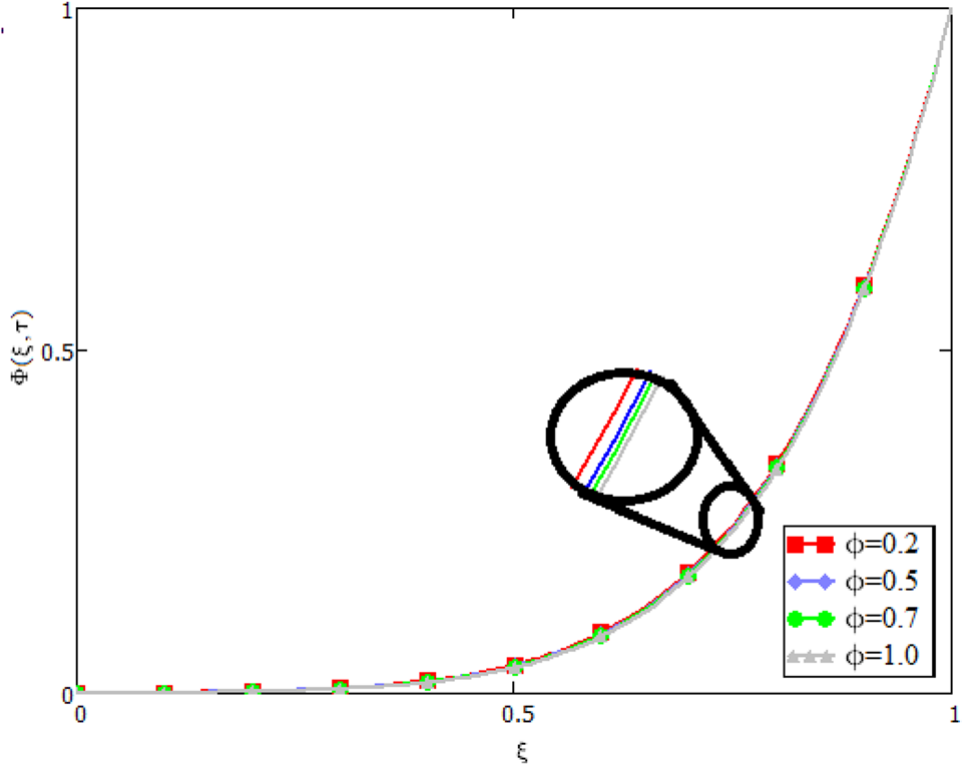
Variation in concentration profile for various values of $\phi$, when $\alpha = 0.5$, $Gr = 0.5$, $Gm = 0.5$,$M = 1$, $\gamma = \frac{\pi }{4}$,$\beta = 1$, $\Pr = 22.64$, and $Sc = 1.9 \times 10^{4} .$

**Figure 12:**
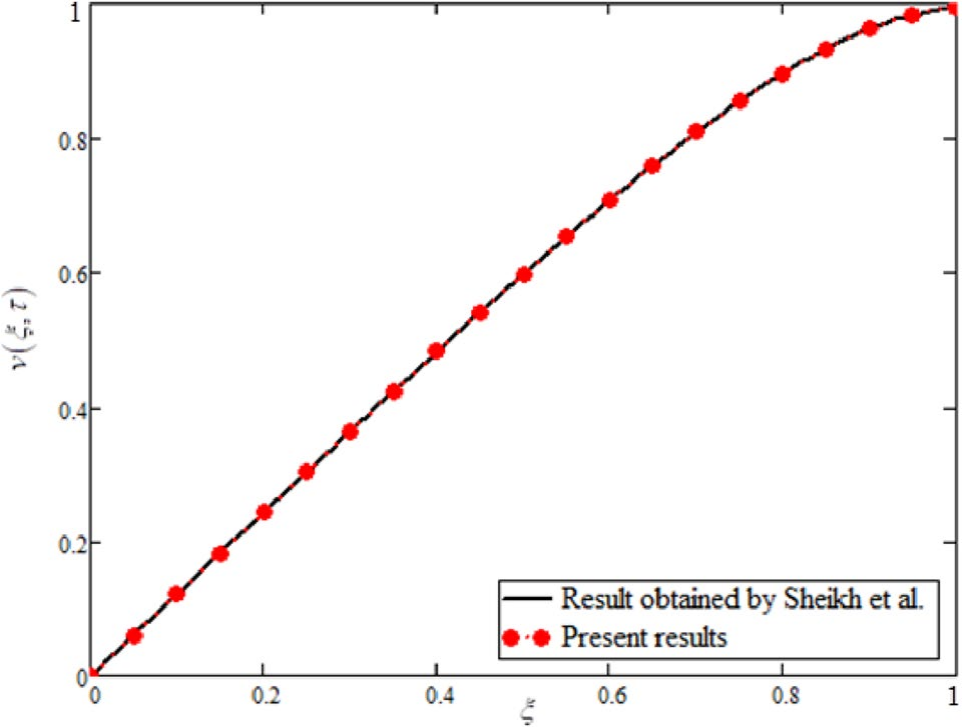
Comparison of obtained results with the results of Sheikh et al^38^, when $\phi = 0$ and $\gamma = 0$.

In this study, the unsteady Casson blood flow with gold nanoparticles between an inclined channel has been discussed. Using the Caputo time fractional operator, the fractional model is developed by transforming the classical model. The exact solutions are achieved by the joint application of the Laplace and Fourier transformations. Some physical parameters that influence a velocity, temperature profile, and concentration profile have been studied extensively. Fractional order modelling is the generalization of classical/integer order models. It is a best tool to include the memory effect, crossover behavior and fractional characteristics. It is also a best tool to best fit the theoretical results with the experimental results, real data and field surveys as it gives us many solutions in the form of variety of integral curves which make it easy to best fit the theoretical results with one of the integral curves with least error. From our graphical results given in Fig. 2, it can be noticed that variation in fractional parameter give us different curves which leads us to best fit the experimental results with our theoretical results. Figure 3 depicts the variance in the velocity profile over a variety of different values of $\phi$. This figure shows that increasing the values of $\phi$ decreases its velocity. The reason for the reduction in velocity is that when $\phi$ rises, the fluid viscosity increases, resulting in the retardation of velocity. Figures 4 and 5 illustrate the effect of the thermal Grashof number $Gr$ and the mass Grashof number $Gm$ on the velocity profile. These graphical representations demonstrate that velocity is an increasing function of these numbers. This is physically accurate since increasing $Gr$ and $Gm$, increasing buoyant forces, which decrease the fluid's viscosity, increasing velocity. Figure 6 shows the profile of velocity for different values of $\beta$ (Casson fluid parameter). Raising the material parameter increases the velocity profile, which depicts the fluid's behavior as Newtonian fluid for $\beta \to \infty$. It's easy to observe in Fig. 7 how the Hartman number influences the velocity profile. It is a relationship between electromagnetic and viscous forces. Lorentz (Flow opposing) forces get stronger as $M$ increases, retarding the velocity. Figure 8 depicts the variation in the temperature profile as the fractional parameter $\alpha$ values change. This is one of the advantages of the fractional derivative; it provides for the analysis of several temperature profiles, as seen in Fig. 2. Figure 9 shows how the volume fraction $\phi$ affects the temperature distribution. It is clear from the figure that heat transfer increases as the values of $\phi$ increase. Higher values of $\phi$ increase the fluid's absorption capacity, and as a result, the fluid temperature increases. The concentration profile with various values of the fractional parameter $\alpha$ is presented in Fig. 10. As indicated in Fig. 2, it has been noted that in the case of concentration profiles, it gives many concentration profiles for studying fluid behavior. Figure 11 is drawn to show the impact of on the concentration profile. The concentration profile is decreased for different values of $\phi$. It may be explained by the fact that viscous forces increase when the concentration profile slows down. Figure 12 illustrates the difference between our results and those of the published studies of Sheikh et al^38^. From the figure we have noticed that by taking $\gamma = 0$ and $\phi = 0$, our results reduced to the solution of Sheikh et al^38^. Furthermore, Table 1 Show the Thermo-physical properties of base fluid(blood) and gold nanoparticles Tables 2 and 3 show variations in the Nusselt and Sherwood numbers for various values $\phi$. It has been discovered that raising the values of $\phi$ by 0.04 percent results a 3.28% increase in heat transfer rate and a 1.626% reduction in mass distribution.

**Table 1:** Thermo-physical properties of base fluid(blood) and gold nanoparticles^8,9,49^.

Material	Symbol	$\rho (Kg/m^{3} )$	$c_{p} (JK/gK)$	$k(W/mK)$	$\Pr$
Blood	–	1050	3617	0.52	22.64
Gold	Au	19,300	129	318	–

**Table 2:** Variation in Nusselt number in response to volume fraction of gold nanoparticles.

$\phi$	$\tau$	$Nu$	$\%$
0	1	0.183	–
0.01	1	0.185	1.092
0.02	1	0.187	2.185
0.03	1	0.188	2.732
0.04	1	0.190	3.825

**Table 3:** Variation in Sherwood number in response to volume fraction of gold nanoparticles.

$\phi$	$\alpha$	$\tau$	$Sh$	$\%$
0	0.2	1	0.129	–
0.01	0.2	1	0.127	0.775
0.02	0.2	1	0.125	1.550
0.03	0.2	1	0.123	3.325
0.04	0.2	1	0.121	3.857

## Conclusion

In the study, a fractional initial and boundary values problem is modeled for the flow of human blood with gold $\left( {Au} \right)$ nanoparticles over an inclined channel. A new approach is used to developed the fractional model. Generalized Fourier’s and Fick’s laws are used to fractionalize the model. Closed-form solutions have been obtained by utilizing the Joint Laplace and Fourier sine transform. Numerous physical parameters have been used to highlight their impact on fluid velocity. The following significant observations have been made from this investigation based on the preceding results and discussion.

- Fick’s and Fourie’s laws are used to transform the time derivative to time-fractional model.
- The variations in all the profiles are shown for differen values of $\alpha$. It is important here to mention that we have different lines for one value of time. This effect is showing the memory effect in the fluid, which cannot be demonstrated from the integer order derivative.
- The used transformation is well suitable for the solution of a fractional model.
- The velocity of the Casson fluid is higher for the grater values of $\beta$, which shows that the fluid will behave like a Newtonian viscous fluid for higher values of $\beta$.
- By increasing the $M$ the velocity is decreases while increasing $Gr$ and $Gm$ the velocity is increasing.
- It is interesting to note that the heat transfer rate of blood is enhanced by 3.825% for gold $\left( {Au} \right)$ nanoparticles.

## Data Availability

All data generated or analyzed during this study are included in this published article.

## Abbreviations

$$u$$: Velocity
$\mu_{nf} e_{ij}$: The $\left( {i,j} \right){th}$ component of deformation rate
$\pi = e_{ij} .e_{ij}$: The product of component of rate itself
$\pi_{C}$: The critical value of this product based on the non-Newtonian fluid
$$\beta$$: Casson parameter
$Gr$: Thermal Grashof number
$Gm$: Mass Grashof number
$$M$$: Hartman number
$$\Pr$$: Prandtl number
$$Sc$$: Schmidt number
$\mu_{f}$: Viscosity of the base fluid $\left( {{\text{Kgm}}^{ - 1} {\text{s}}^{ - 1} } \right)$
$K_{f}$: Thermal conductivity of the base fluid $\left( {{\text{Wm}}^{ - 1} {\text{K}}^{ - 1} } \right)$
$K_{nf}$: Thermal conductivity of nanofluids $\left( {{\text{Wm}}^{ - 1} {\text{K}}^{ - 1} } \right)$
$K_{s}$: Thermal conductivity of nanoparticles $\left( {{\text{Wm}}^{ - 1} {\text{K}}^{ - 1} } \right)$
$\mu_{nf}$: Dynamic viscosity of nanofluids $\left( {{\text{Kgm}}^{ - 1} {\text{s}}^{ - 1} } \right)$
$\sigma_{f}$: Electrical conductivity of base fluid $\left( {{\text{s}}^{3} {\text{A}}^{2} {\text{m}}^{ - 1} {\text{kg}}^{ - 1} } \right)$
$\sigma_{nf}$: Electrical conductivity of nanofluids $\left( {{\text{s}}^{3} {\text{A}}^{2} {\text{m}}^{ - 1} {\text{kg}}^{ - 1} } \right)$
$\sigma_{s}$: Electrical conductivity of nanoparticles $\left( {{\text{s}}^{3} {\text{A}}^{2} {\text{m}}^{ - 1} {\text{kg}}^{ - 1} } \right)$
$$\alpha$$: Fractional parameter
$\rho_{s}$: Density of the solid particles $\left( {{\text{kgm}}^{ - 3} } \right)$
$\rho_{f}$: Density of the fluid $\left( {{\text{kgm}}^{ - 3} } \right)$
$\rho_{nf}$: Density of nanofluids $\left( {{\text{kgm}}^{ - 3} } \right)$
$\phi$: Nanoparticles volume fraction
$cp$: Specific heat capacity
$$T$$: Temperature $$\left( K \right)$$
$\beta_{nf}$: Thermal expansion coefficient
$\beta_{f}$: Thermal expansion coefficient of base fluid $\left( K \right)$
$\beta_{s}$: Thermal expansion coefficient of nanoparticles $\left( {K^{ - 1} } \right);$
